# Immunotherapy of Malignant Disease Using Chimeric Antigen Receptor Engrafted T Cells

**DOI:** 10.5402/2012/278093

**Published:** 2012-12-09

**Authors:** John Maher

**Affiliations:** ^1^CAR Mechanics Group, Department of Research Oncology, King's Health Partners Integrated Cancer Centre, King's College London, Guy's Hospital Campus, Great Maze Pond, London SE1 9RT, UK; ^2^Department of Immunology, Barnet and Chase Farm Hospitals NHS Trust, Barnet, Hertfordshire EN5 3DJ, UK; ^3^Department of Clinical Immunology and Allergy, King's College Hospital NHS Foundation Trust, Denmark Hill, London SE5 9RS, UK

## Abstract

Chimeric antigen receptor- (CAR-) based immunotherapy has been under development for almost 25 years, over which period it has progressed from a new but cumbersome technology to an emerging therapeutic modality for malignant disease. The approach involves the genetic engineering of fusion receptors (CARs) that couple the HLA-independent binding of cell surface target molecules to the delivery of a tailored activating signal to host immune cells. Engineered CARs are delivered most commonly to peripheral blood T cells using a range of vector systems, most commonly integrating viral vectors. Preclinical refinement of this approach has proceeded over several years to the point that clinical testing is now being undertaken at several centres, using increasingly sophisticated and therapeutically successful genetic payloads. This paper considers several aspects of the pre-clinical and clinical development of CAR-based immunotherapy and how this technology is acquiring an increasing niche in the treatment of both solid and haematological malignancies.

## 1. Introduction to Chimeric Antigen Receptor Technology

Tumour immunotherapy is one of the oldest branches of clinical immunology and has a long but checkered history. The overriding goal is to deploy the multiplicity of available immune effector mechanisms against tumour cells, but not against healthy counterparts. Unfortunately however, several obstacles render this a very difficult goal. Although hundreds of so-called tumour antigens have been identified, these are generally derived from self and thus are poorly immunogenic. Furthermore, tumours use several mechanisms to render themselves hostile to the initiation and propagation of immune attack. These immune subversive strategies include reduced expression of HLA molecules and target antigens coupled with the establishment of a microenvironment in which inhibitory cytokines and leukocytes abound (recently reviewed in [1]). Indeed, cancer cells can even dedifferentiate to evade detection in response to inflammatory cues provided by tumour-specific T cells [2]. Consequently, it is not surprising that attempts to harness tumour-specific T cells using a succession of vaccination-based approaches have not achieved striking success [3]. 

Recent developments using genetically enhanced T cells have led to renewed optimism in the quest to launch immune attack against malignant disease. One increasingly prominent technology in this arena involves the use of the so-called chimeric antigen receptors, or CARs. These bespoke fusion receptors are engineered as chimeric cDNAs and couple the recognition of a designated molecular species on the tumour cell surface to the delivery of a tailored T cell activating signal. A defining property of this technology is the fact that, unlike *αβ* T cell receptors (TCR), antigen recognition by CARs is direct and thus is generally not restricted by polymorphic presenting elements such as human leukocyte antigens (HLAs). Advantages of such an immunotherapeutic targeting strategy are threefold. First, since function is not dependent upon HLA status, the same CAR-based approach can in principle be used in all patients in whom tumours express the target of interest. Second, corruption of antigen processing and presenting machinery is a common attribute of transformed cells and may facilitate immune escape. However, this affords no protection against CAR-engineered T cells. Third, a range of macromolecules can be targeted using this system, including proteins, carbohydrates, and glycolipids. Adding further flexibility to this approach, intracellular antigens can be targeted using CARs that recognize defined HLA-peptide combinations [4–6].

The birth of CAR technology occurred 25 years ago when it was shown that antibody variable light (*V*_*L*_) or heavy (*V*_*H*_) gene segments can transfer specificity for native antigen, when substituted for the corresponding elements within a TCR *αβ* heterodimer [7]. It was Eshhar who realized the translational potential of such non-HLA-restricted T cell recognition [8, 9]. Because T cell activation is coupled to antibody-like recognition, he coined the term T-body to describe this technology. In its early stages, the approach was cumbersome since two chimeric genes (comprising *V*_*L*_ and *V*_*H*_-directed specificity) needed to be delivered together in order to redirect specificity for antigen. This challenge was overcome with the use of linker sequences that permit *V*_*L*_ and *V*_*H*_ modules to self-associate, thereby creating a single chain variable fragment (scFv). As a result, T cell specificity could now be effectively redirected using a single receptor dimer [10]. 

## 2. Structural Refinement of Chimeric Antigen Receptors: Evolution through the Generations

**Figure 1:**
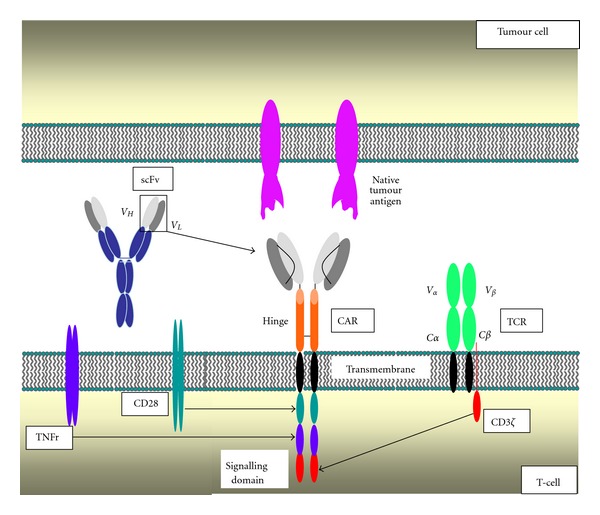
Generic structure of a chimeric antigen receptor (CAR). These fusion receptors comprise a targeting moiety (in this example, an antibody-derived single chain antibody (scFv)), coupled via a hinge and transmembrane element to a bespoke modular signalling domain. This example shows a “third generation” CAR in which signalling is provided by CD3*ζ* together with costimulation provided by CD28 and a tumour necrosis factor receptor (TNFr), such as 4-1BB or OX40.

The overall structure of a CAR consists of four elements that are joined in series (Figure 1). Antigen engagement is achieved by a targeting domain, which is commonly separated from a membrane-spanning element by an extracellular hinge/spacer segment. Upon cross-linking by target antigen, signals are transmitted to engineered cells via the CAR endodomain. Each of these four elements has been subjected to careful refinement and some of the most salient developments are summarised briefly below.

Antigen targeting by CAR molecules most commonly involves the use of scFv that have been assembled from monoclonal antibodies. However, several alternative targeting moieties may also serve this purpose. These include ligands [11, 12], peptides [13], chimeric ligands [14], receptor derivatives [15, 16], and single domain antibodies [17]. Several molecules that are commonly expressed by diverse solid and haematological malignancies have been shown to be amenable to CAR-directed targeting (summarised in Table 1). In addition to antigen-specific approaches, two “universal” CAR systems have recently been described. These generic CARs contain avidin [18] or antifluorescein isothiocyanate (FITC) scFv [19, 20], enabling their use in conjunction with separate targeting moieties that have been biotinylated or conjugated to FITC, respectively. 

Several factors may influence the specificity and selectivity of tumour epitope engagement by the targeting domain. Unlike *αβ* TCR, antigen engagement by CARs generally involves a high affinity interaction. However, increasing affinity beyond a certain threshold does not increase targeting efficiency, but may in fact be counterproductive since it renders antigen^lo^ healthy cells also amenable to recognition [21]. The outcome of this interaction is further complicated by molecular heterogeneity of the target epitope, even within the same cell. A good example of this principle is the MUC1 mucin, in which several distinct glycoform epitopes are found, each of which binds with differing efficiency to scFv-derived CARs [22]. A further issue that may compromise function of the CAR targeting domain is its immunogenicity. Earlier fusions commonly contained murine scFv sequences that elicited the formation of blocking antibody responses [23, 24]. More recently, there has been a move towards the use of humanized or fully human molecules. While this may ameliorate the problem it is unlikely to eliminate it completely owing to the presence of idiotype sequences and fusion junctions between CAR components. 

The second element within a CAR molecule is the spacer/hinge domain, which serves to separate the targeting moiety from the T cell plasma membrane [25]. Recently, it has become clear that the spacer can also profoundly influence CAR function. When CARs engage membrane-proximal epitopes, T cell activation is potentiated by the inclusion of a spacer element, which provides the necessary “reach” to facilitate target engagement. By contrast, the inclusion of spacer domains may impair function when targeting epitopes lie far from the target cell surface [26]. These observations suggest that there is an optimum distance between target and T cell membranes in order to achieve effective CAR-mediated function. In keeping with this, it has been shown that strength of CAR signalling is less when cognate epitope lies far from the target cell membrane. Indeed such lowered activity may be exploited to enable CAR-engineered T cells to discriminate more effectively between antigen^hi^ tumour cells and antigen^lo^ healthy cells [27]. Complex targets such as MUC1 may also impose considerable size and glycosylation-related steric hindrance. This challenge has been circumvented using a flexible and elongated spacer/hinge, such as that found in IgD antibody [22]. The spacer/hinge can also influence the interaction between CAR-engineered T cells and other elements of the immune system. For example, human IgG-derived Fc sequences are commonly used owing to their stabilizing effects on CAR expression. However, these elements can also activate innate (Fc receptor-expressing) immune cells, an outcome that can be abrogated through appropriate mutation of this element [28].

Similarly to the hinge, the CAR transmembrane domain is also considered to serve primarily a structural function. Commonly used sequences are derived from T cell molecules such as CD4, CD8, or CD28. Once again however, recent evidence indicates that transmembrane sequences may also influence CAR function. For example, fusion molecules that incorporate a CD3*ζ* transmembrane domain associate with elements of the endogenous TCR/CD3 complex, leading to heightened sensitivity of T cell activation [29].

Delivery of signals by CAR molecules is achieved by the endodomain. This element has been manipulated extensively in an attempt to optimise function of engineered cells. Early CAR designs contained endodomains that were selected to provide signals that mimic those delivered naturally by the TCR/CD3 complex. These co-called first generation CARs most commonly employ either intracellular sequences derived from CD3*ζ* or from the *γ* subunit of the high affinity receptor for IgE, Fc*ε*R1. The CD3*ζ* subunit provides a sufficient signal to mimic that provided by the complete CD3 complex [30] and furthermore has consistently proven superior as a source of signal 1, when compared to Fc*ε*R1-*γ* [31]. To refine function, CARs were next engineered that provide costimulatory type signals to T cells (“signal 2” [32]). This was a logical progression since the majority of tumour types does not express co-stimulatory ligands. In order to enhance potency, second generation CAR designs were first developed by Finney et al. who made an in-series fusion of CD28 and CD3*ζ* endodomain sequences [33]. Proof of principle for the advantage of this design was demonstrated using Jurkat T cells and confirmed thereafter using primary human [34] and murine T cells [35, 36]. While cytolytic activity was generally not enhanced [34, 37], the key advantage conferred by the use of second generation CARs was the induction of IL-2 secretion and T cell proliferation upon CAR cross-linking. More recently, several alternative second generation CAR designs have been described in which “signal 2” was provided by other co-stimulatory receptors, including ICOS (inducible costimulatory), OX40 (CD134 [38, 39]), 4-1BB (CD137 [38–40]), CD27 [41], DAP10 [39], or 2B4 (CD244; [42]). When compared to CARs that employ CD3*ζ* alone, improved function was demonstrable with all such second generation designs. However, notable differences were also apparent between constructs. Antigen-triggered production of IL-2 is generally maximal using the CD28-CD3*ζ* configuration [39]. By contrast, cytotoxicity was greatest in one study with the ICOS-CD3*ζ* combination [38] and recent evidence indicates that this combination may promote the sustained persistence of Th17-type CAR engineered T cells [43]. Nonetheless, a clear consensus from different studies is difficult to obtain. For example, inclusion of CD28 sequences has been reported to render cells resistant to regulatory cytokines and T cells in some studies [44, 45]. However, in other model systems, the greater production of IL-2 by CD28-CD3*ζ* CARs leads to enhanced infiltration by regulatory T cells, leading ultimately to poorer CAR-directed antitumour activity [46]. Multifunctionality of cytokine production has been reported to be greatest with CAR containing CD3*ζ* and 4-1BB (alone or together with CD28) [37]. Once again however, this has not been universally observed [39]. Intriguingly, in some studies, second generation CARs that combine 4-1BB and CD3*ζ* exhibit antigen-independent activity. This finding was associated with improved engraftment and antitumour activity *in vivo*, when compared to CD3*ζ* or CD28-CD3*ζ* designs [47]. Improved antitumour function has been observed in pre-clinical *in vivo* models where several distinct second generation CARs have been compared to first generation counterparts [39, 41]. Improved *in vivo* survival of T cells has been observed where CD28-*ζ*, CD27-*ζ*, or 4-1BB*ζ* designs have been tested in mice [37, 41, 48], a finding that has also been observed in man [49–51]. Resistance to activation-induced cell death and improved T cell survival (mediated via activation of AKT/mammalian target of rapamycin and antiapoptotic Bcl2 family members) may underlie this observation. 

In addition to the inclusion of co-stimulatory sequences in series with CD3*ζ*, a number of alternative approaches have been developed to boost the function of first generation tumour-targeted CARs. Wilkie et al. have recently coexpressed two CARs in the same T cell population with the goal of providing signals 1 and 2 to these cells upon simultaneous engagement of both target antigens. In that study, fusion receptors were directed against two breast cancer-associated targets, namely, MUC1 and ErbB2. *In-vitro *evidence of synergistic T cell activation was obtained upon engagement of both targets [52]. However, formal comparison of delivery of costimulation *in cis* (using second generation CARs) compared to *in trans* has not been performed as yet. A second alternative approach involves the coexpression of a constitutively active Akt mutant in CAR-engineered T cells [53]. Increased T cell proliferation, cytokine production, Granzyme B expression, and tumour cell cytotoxicity were observed, accompanied by reduced apoptosis and resistance to suppression by regulatory T cells [53]. Although autoimmune toxicity may be a risk associated with this strategy, this could be addressed in a number of ways, for example, through the co-expression of a suitable suicide gene (see below). A third system that has recently been described to boost CAR function entails the co-expression of a mutated form of LAT (linker for activation of T cells) that is resistant to degradation. Compared to T cells that express CAR alone, this modification also results in enhanced antitumour cytolytic activity, accompanied by increased cytokine production. [54]. 

Following the successful development of second generation CARs, it was natural that investigators would go on to develop fusions that can deliver more than one type of costimulatory signal. Several such “third generation” CARs have been described in which CD3*ζ* has been coexpressed with p56 lck + CD28 [55]; OX-40 + CD28 [22, 56, 57]; or 4-1BB + CD28 [22, 37, 58, 59]). A possible contributory mechanism to superior function of these more complex CARs stems from the fact that CD28-CD3*ζ* second generation CARs elicit greater production of the inhibitory cytokine, IL-10. However, inclusion of OX40 [56] or 4-1BB [47] sequences can reduce this unwanted effect while preserving or enhancing the production of proinflammatory cytokines. Some recent comparative studies have demonstrated an advantage for third generation CARs (e.g., 4-1BB plus CD28) over fusions that provide co-stimulation from either 4-1BB or CD28 alone [47, 58]. However, definitive conclusions are difficult to make once again since many studies have not included a comparison of all available second and third generation CARs using* in vivo* models. This is important since it is often observed that *in-vitro* comparisons do not accurately predict differences that may be observed upon *in vivo* testing. In a related development, an interesting alternative strategy to deliver dual co-stimulation involves the co-expression of 4-1BB ligand and CD80 in T cells together with a first generation CAR. Using this approach, striking enhancement of anti-tumour activity has been demonstrated in comparison to cells that receive only one form of co-stimulatory signal [60] although there are concerns that constitutive co-stimulation may favour autoimmune toxicity [61–63]. 

## 3. Host Cells for CAR-Based Immunotherapy

In the majority of studies, CARs are expressed in autologous patient-derived T cells. However, function of CAR-based fusion receptors has been demonstrated in other leukocyte populations as summarised in Table 2. Several groups have shown that natural killer cells can be retargeted effectively with CD3*ζ*-based fusions. Further enhanced anti-tumour activity is observed if 4-1BB [64] or 2B4 [65] sequences are also incorporated into the CAR endodomain. Using this approach, NK cells can be engineered to destroy leukaemic cells that are otherwise naturally resistant to these effectors. Although NK cells account for only ≤10% of circulating mononuclear cells, they can be expanded using K562 feeder cells that express cytokines (e.g., membrane anchored IL-15 and/or IL-21) and 4-1BB ligand [64, 66]. Clinically applicable processes to achieve this have now been refined using a K562 master cell bank manufactured under good manufacturing process (GMP) [67]. Gamma delta T cells can similarly be retargeted effectively using CAR-based technology [68]. Once again, although *γδ* T cells represent only ≤5% of circulating mononuclear cells, they can be expanded *ex vivo* using clinical available aminobisphosphonates such as zoledronic acid, making clinical translation of this approach conceivable.

Use of patient-derived cells imposes constraints on the practicalities of cell product manufacture. However, allogeneic T cells are generally not suitable for this purpose since they could elicit severe graft versus host disease (GvHD), particularly if infused into immunosuppressed (e.g., lymphodepleted) recipients. To circumvent this, zinc finger nucleases have been delivered transiently to T cells in order to mutate the *α* and *β* subunits of the endogenous TCR complex so they are no longer expressed [69]. By this means, it can be envisioned that banks of CAR-engineered T cells could be produced for widespread application in allogeneic recipients, without risk of graft versus host disease. 

## 4. Optimising the Tumour Microenvironment for CAR-Based Immunotherapy

The immune inhibitory nature of the tumour microenvironment constitutes a key hurdle to the successful implementation of CAR-mediated immunotherapy. Several myeloid (e.g., myeloid-derived suppressor cells; some tumour-associated macrophage populations) and lymphoid cell populations (e.g., regulatory T cells) are commonly found at that location and produce a diversity of factors (e.g., transforming growth factor-*β*, IL-10, prostaglandin E2, PD-L1 (programmed death 1 ligand 1)) that conspire to inhibit immune-mediated attack. 

Several approaches have been used or are in development in order to render the tumour microenvironment more favourable to CAR-based immunotherapy. Use of preemptive chemotherapy to achieve either lymphodepletion and/or myeloablation has led to a marked improvement in the efficacy of adoptive immunotherapy using *ex vivo* expanded tumour-infiltrating lymphocytes [70, 71]. Such conditioning therapy approaches are now beginning to impact favourably upon the use of CAR-engineered cells. However, not all patients are sufficiently fit for such treatment. Furthermore, there may be a greater potential for toxicity with such combined therapies, as discussed further below. Consequently, it is desirable to develop more refined approaches to achieve this goal if possible.

Immune competent murine models provide an opportunity to address the role of the tumour microenvironment and to develop strategies to mould the microenvironment so that is it more conducive to anti-tumour immune responses. An excellent example of this principle involves the use of T cells engineered to express a CAR in which NKG2D is coupled to CD3*ζ* [72]. The NKG2D receptor engages several stress ligands that are commonly upregulated on tumour cells. Consequently, NKG2D-targeted CARs can effect the destruction of tumour cells. Intriguingly however, these engineered T cells also exert profound effects upon elements of the tumour microenvironment. Since Foxp3^+^ regulatory T cells may express stress ligands, they are also amenable to destruction by NKG2D-targeted effector T cells [72]. Since the engineered T cells produce cytokines, notably granulocyte macrophage colony-stimulating factor (GM-CSF) and interferon (IFN)-*γ*, this promotes the recruitment tumour-associated macrophages in which antigen-presenting function and tumour lytic activity are both enhanced [72, 73]. As a result, an endogenous anti-tumour immune response can be activated within the reconfigured tumour microenvironment [72].

An alternative approach that may be used to modify the tumour microenvironment involves the delivery of IL-12 to this location. When tumour-specific T cells are modified to secrete this heterodimeric cytokine, anti-tumour function is markedly enhanced such that small numbers of infused cells can achieve impressive anti-tumour function when infused into lymphodepleted hosts [74]. More recently, CAR-engineered T cells have been engineered to produce IL-12 either in a constitutive or inducible manner within the tumour microenvironment, leading to impressive enhancement of anti-tumour activity [75–77]. Several mechanisms appear to account for this observation. First, IL-12 enhances T cell and NK-cell function, in part through increased perforin and granzyme expression, accompanied by lowered IL-2 production and relative insensitivity to the suppressive effects of regulatory T cells [76]. Second, IL-12 recruits and activates innate immune cells (e.g., NK cells, macrophages, and NK-T cells) [77]. Third, IL-12 reprogrammes the function of several myeloid cell populations within the tumour microenvironment, effectively converting them from immunosuppressive to immunostimulatory cells [78]. Indeed, this effect may also contribute to the destruction of antigen null tumour cells by macrophages [77]. Fourth, IL-12 exerts antiangiogenic effects within tumours. The potentiating action of constitutive IL-12 expression upon CAR T cell function was sufficiently potent in one study that the need for lymphodepletion was circumvented by this approach [76]. Clinical trials are ongoing in patients with melanoma in which IL-12 is constitutively expressed in tumour-infiltrating lymphocytes prior to *ex vivo* expansion and infusion (http://clinicaltrials.gov/ Identifier: NCT01236573). 

## 5. Directing T-Cell Trafficking

Poor T cell trafficking to tumour deposits is a major limitation to the effectiveness of adoptive immunotherapy using CAR-engineered T cells [79]. Virtually all clinical trials entail the intravenous delivery of these cells. When delivered in this manner, cells become physically stuck in the lungs for several hours [23]. Thereafter, redistribution of the bulk of the infused cells to the liver and spleen is observed while some cells can also be visualised in lymph nodes. Clinical studies in haematological malignancies have shown that some CAR-engineered T cells can traffic to tumour deposits [49, 51, 80]. Nonetheless, there is a clear rationale to improve the efficiency of this process.

To address this, a number of experimental strategies are under investigation. Overproduction of several molecules with chemotactic properties, notably chemokines, is a feature of many tumour types. To exploit this, investigators have expressed chemokine [81–83] and other cytokine receptors [84] in engineered T cells in order to guide their chemotactic migration. Alternatively, bone marrow homing can be enhanced with total body irradiation or cyclophosphamide, both of which stimulate the production of chemoattractants such as stromal-derived factor-1 [85]. A third strategy under increasing study involves the enzymatic modification of cells with fucosyltransferases, enabling cells to engage E-selectin, which is constitutively expressed by bone marrow endothelium. By this means, enhanced bone marrow trafficking of various haematopoietic cells can be achieved [86, 87]. 

## 6. The T cell Survival Problem

Another key obstacle to the efficacy of adoptive T cell immunotherapy using CAR-engineered T cells has been poor survival of the infused cells in patients. This limitation was consistently observed in early clinical studies in which first-generation CARs were used to treat patients. However, several recent developments have begun to address this challenge. A major advance was the incorporation of lymphodepleting conditioning regimens prior to infusion of CAR-mediated T cells. Such preparative conditioning had earlier been shown to improve the efficacy of immunotherapy using tumour-infiltrating lymphocytes [70]. Lympho- or myelodepletion removes unwanted suppressive cellular populations such as regulatory T cells and several suppressive myeloid cell types. Furthermore, it creates space for the expansion of infused cells, eliminates cytokine sinks and thus allows the infused cells to benefit from increased production of and access to homeostatic cytokines (e.g., IL-7 and IL-15) and other cytokines (e.g., IFN-*γ* and IL-12) [76]. Most recent clinical trials using CAR-engineered T cells incorporate a lymphodepletion step and this is very likely to have contributed to some impressive preliminary clinical responses, as described further below. However, not all patients are sufficiently fit to tolerate such profoundly immunosuppressive treatment. Furthermore, if excessive activation of infused cells occurs in the lymphodepleted host, unacceptable toxicity and, on occasion, cytokine storm has been the result [88–90]. 

A second approach to improve *in vivo* T cell survival entails the delivery of CAR transgenes to virus-specific T cells. Proof of principle for the effectiveness of this strategy has been demonstrated using T cells specific for influenza [91], Epstein Barr virus (EBV) [92–95], cytomegalovirus (CMV) [93], and a combination of viral antigens [96]. Clinical trials using this approach are ongoing in multiple centres and are discussed in greater detail below.

A third strategy to improve T cell survival *in vivo* involves the judicious provision of cytokine support. Most commonly, IL-2 is used for this purpose and when administered at low-dose, it has been shown to prolong the *in vivo* persistence of CAR-targeted T cells [97]. However, IL-2 is not selective for the infused T cells and is toxic when administered in high doses, mediated in part through the induction of systemic autophagy [98]. Gene transfer may be employed to deliver autocrine cytokine support to T cells, leading to enhanced longevity and anti-tumour activity in preclinical models [99]. However, early clinical experience with such a strategy has proven disappointing [100]. To harness selective cytokine support for CAR-based immunotherapy, several experimental approaches have been developed. For example, chimeric GM-CSF/IL-2 receptor subunits have been expressed in order to enable activated T cells to benefit from autocrine stimulation by GM-CSF [101]. Similarly, enforced expression of IL-7 receptor *α* in infused cytotoxic T cells has been used to restore their responsiveness to this cytokine [102]. Translation of such an approach is supported by the availability of clinical grade IL-7 for administration in man. An alternative strategy is to render T cells responsive to tumour-associated cytokines. Several tumours overproduce colony-stimulating factor-1 (CSF-1) and this has been harnessed to support T cell survival and migration by expression of the CSF-1 receptor in T cells [84]. In a related development, CAR-engineered T cells have been engineered to co-express a chimeric cytokine receptor that converts the binding of IL-4 (a weak T cell mitogen) to delivery of a potent IL-2/15-type growth signal [103]. This system has been used to enrich CAR-modified T cells *ex vivo *[14] and also has the potential to be applied in the treatment of malignancies such as prostate cancer where IL-4 is overproduced in the tumour microenvironment [104]. A further elegant system that may find increasing application involves the use of immunocytokines that target cytokine delivery to tumour cells. Such an approach has been used to target IL-2 to B cells and thereby improve the longevity and anti-tumour activity of CD19-targeted T cell immunotherapy [105].

An increasingly studied device to improve T cell engraftment *in vivo* involves the delivery of CAR transgenes to more immature or less differentiated T cell populations. Although these cells may exhibit less effector function when tested *in-vitro*, they are more long lived and have greater capacity for *in vivo* survival and proliferation. To capitalise on this, a clinical grade process has recently been described to deliver a CD19-specific CAR to virus-specific (EBV/CMV) central memory T cells [93]. Such an approach may find particular application in the context of allogeneic haematopoietic stem cell transplantation for aggressive B cell malignancy where the infused cells may consolidate remission and protect from viral reactivation while conferring lowered risk of inducing graft versus host disease. Building on this, the recent description of a human stem cell memory T cell compartment provides an attractive substrate for further refinement of this strategy. Alternatively, several strategies have been proposed to increase T cell “stemness” during the manufacture of cell products for clinical use [106]. 

## 7. Gene Delivery Systems

In the majority of clinical and preclinical research involving CAR-based immunotherapy, retroviral or lentiviral vectors are used to effect gene transfer. The ability of such vectors to integrate into the host cell chromosome raises the possibility of insertional mutagenesis and oncogene activation [107]. In keeping with this, acute leukaemia has developed in 5 of 19 children treated with gene therapy for X-linked severe combined immunodeficiency in France and the UK. Furthermore, insertion-related overexpression of a small number of genetic loci and resultant myelodysplastic syndrome has also been observed following retrovirus-mediated haemopoietic stem cell gene therapy for X-linked chronic granulomatous disease [108, 109]. 

By contrast to haematopoietic stem cells, recent studies indicate that mature T cells are highly resistant to transformation by gamma-retrovirus transduction. Occasionally, immortalization of T cells has been achieved with such vectors [110]. Even under these extreme circumstances however, such T cells are not tumorigenic *in vivo*. Data from several sources indicates that gammaretroviral vectors do not elicit clinically significant genotoxicity when used to deliver therapeutic genes to T cells. To date more than 200 patients have received genetically modified T cells in diverse clinical trials [15, 111–116], notably patients with haematological malignancy and HIV infection. These transduced populations maintain gene expression profiles, phenotype, cytokine responses, and TCR diversity *in vivo,* in the absence of clonal selection, immortalization, or other integration-related toxicity up to 11 years after administration [111–113]. 

In parallel activity, several alternative gene transfer systems are under development. Transient gene expression can be achieved using plasmid [38] or RNA electroporation [117–120]. Such methods are less expensive to develop for clinical purposes. Alternatively, transposons are capable of integrating into the human genome and stably expressing transgenes and are also less expensive and easier to manufacture than viral vectors [121, 122].

## 8. Cell Expansion Systems

Manufacture of genetically engineered cell products for clinical use requires GMP systems that control all operations, from the receipt of raw materials of adequate quality, through to production, (re)packaging, (re)labelling, internal quality control, control of release, storage, stability, and, distribution of cell products. Documentation (e.g., quality manual, standard operating procedures, and batch manufacturing records) and document control are key elements in the achievement of a quality management system. Open manipulations during the manufacture of cell products must be conducted within isolators that are housed in clean room facilities. The ideal manufacturing system is closed from the point of phlebotomy to the point of reinfusion of cells into patients. To minimize the need for isolator use, there is an increasing interest in the development of closed manufacturing processes whereby cells are maintained in clinical grade gas-permeable bags which can be joined to feeding bags using closed welder/sealer systems. Sampling can also be performed using such devices, allowing cell count and culture pH to be checked, again without the need to introduce the cultured cells into an isolator. 

Activation and expansion of T cells is commonly achieved using anti-CD3 antibody or beads that are coated with this agent, alone or in combination with CD28 [123]. Alternatively, feeder cell expansion systems have been developed, for example, using HLA class I^NEG^ K562 immortalised leukaemia cells. These cells have been engineered to coexpress Fc*γ* receptors (allowing loading of CD3 antibody) together with a range of costimulatory ligands and/or cytokines, thereby allowing the expansion of either T cells or NK cells [67, 124]. Other clinical grade processes have been developed to allow the expansion of virus-specific T cells, which are commonly used as hosts for CAR-based immunotherapy. In addition to static cell expansion systems, bioreactors may alternatively be used to propagate engineered T cells at higher density, a process that is facilitated by continuous perfusion of the cultures [123].

## 9. Considerations regarding Toxicity and Suicide Genes

The majority of CARs is targeted against self-antigens and thus it is important to consider issues of potential acute and chronic toxicity and how these may be mitigated. On-target off-tumour toxicity is a clear risk and is described further in the sections that summarise clinical experience with this technology. Pre-clinical studies have demonstrated that it is possible to target self-antigens safely while propagating anti-tumour attack using CAR-engineered T cells. For example, ErbB2-specific CAR^+^ T cells have been used to control tumours in ErbB2 transgenic mice without autoimmunity, even when administered following preparative lymphodepletion [125]. Similar results were obtained using CARs targeted against carcinoembryonic antigen (CEA), although autoimmune toxicity was unmasked following myeloablative conditioning [126]. Similar dose-limiting toxicity has been observed in clinical studies involving patients with colorectal cancer who were lymphodepleted and then received systemically administered T cells that had been retargeted with a CEA TCR [127].

To reduce against the risk of chronic toxicity, one of a number of suicide genes may be incorporated into the vector. In this regard, the herpes simplex virus thymidine kinase (HSV-TK) gene has the advantage of having the longest track record of testing, including extensive experience of clinical use. The HSV-TK protein phosphorylates ganciclovir, which enables its incorporation into DNA. Since chain elongation is prevented as a result, this leads to cell death. Ganciclovir is not toxic to cells that do not express HSV-TK since it has a 1000-fold lower affinity for the eukaryotic enzyme. However, use of HSV-TK as a suicide system has a number of limitations. Since it is of viral origin, HSV-TK is immunogenic [128] and can accelerate loss of transduced T cells *in vivo*. This may be less problematic if patients are profoundly immune compromised (e.g., following lymphodepletion or soon after a stem cell transplant) [111]. A further difficulty with HSV-TK is the fact that it acts as a cell cycle-dependent toxin and thus it may be less effective in slowly dividing cells.

In light of these shortcomings, the use of an alternative suicide gene such as inducible caspase-9 is potentially an attractive solution [129]. Caspase 9 is an initiator of apoptosis whose activity is triggered upon dimerisation. In the inducible approach, caspase 9 is fused to a dimerisation domain derived from human FK506 binding protein. As a result, chemical inducers of dimerisation that are derived from FK506 may be used to induce the selective dimerisation of this fusion protein, leading to the induction of apoptosis. In principle, this approach should be minimally immunogenic since all constituents of the fusion gene are of human origin. However, this system requires access to a nonlicensed pharmaceutical agent that has undergone limited clinical testing [130]. A related approach that may also be worthy of testing involves the use of tamoxifen-regulated caspase proteins. These operate on a broadly similar principle and consist of a mutated oestrogen receptor dimerisation domain that binds tamoxifen (but not oestrogen) and which can be coupled to a selected caspase [131].

A third strategy involves the expression of CD20 in engineered T cells. As a consequence, cells should be rendered amenable to elimination using a clinical grade depleting CD20 antibody (e.g., rituximab). A further advantage of this system is the fact that a minimal rituximab epitope has recently been defined [132, 133]. The main disadvantage that can be envisioned using this approach is the fact that healthy B cells would also be eliminated although this generally does not result in alternation of serum immunoglobulin profiles unless rituximab treatment is repeated. However, this system has not been studied in the context of CAR-based immunotherapy as yet.

While the foregoing approaches may prove useful in controlling chronic toxicity, it is by no means certain that activation of a suicide system could achieve any meaningful impact in dealing with acute cytokine storm. In that setting, standardised management protocols have not been developed, although it is reasonable to propose that care should be coordinated at an early stage by an intensive care physician. In addition to resuscitative measures, treatment may involve the use of high dose corticosteroids and a biological agent [134], although firm evidence in support of any specific choice is lacking. 

## 10. Considerations regarding Route of Administration of CAR-Engineered T cells

Virtually all clinical trials of CAR^+^ T cells involve the intravenous (IV) route of administration. When delivered in this manner, cells traffic via the lungs and then redistribute to liver and spleen [23]. Notably however, pulmonary and hepatic toxicity has both been observed in clinical studies using this technology, as is discussed further below. Moreover, symptomatic T cell persistence in the lungs is more prolonged if cells are highly activated [23], as is commonly the case using cell manufacturing protocols. One approach that may circumvent the risk of toxicity when targeting self-antigens entails the intratumoral delivery of T cells. In murine pre-clinical models, human T cells traffic following IV delivery in a very similar manner to that observed in man. Cells become physically stuck in the lungs for several hours and thereafter migrate to the liver, spleen, and lymph nodes [79]. By contrast, when delivered using a regional approach (e.g., subcutaneous, intraperitoneal, or intratumoral), injected T cells largely remain at the site of injection with some local diffusion evident over the following days [79]. Recently, use of the intratumoral route of delivery has been shown to achieve impressive efficacy while preventing autoimmune toxicity in an orthotopic model of CEA^+^ pancreatic cancer, established in CEA transgenic mice [135]. Similarly, intracerebral delivery may be used to achieve this goal in mice engrafted with glioblastoma xenografts [136].

## 11. Imaging of CAR T Cells **In Vivo**

Refinement of CAR-based immunotherapy is greatly facilitated by the use of imaging systems to track the migration, biodistribution, and longevity of adoptively infused T cells *in vivo*. Bioluminescence imaging provides a convenient, sensitive, and high-throughput imaging modality [105, 137], although it is not suitable for clinical translation. Alternatively, single photon emission computed tomography (SPECT) has been used to track human CAR-engineered T cells that had been passively labelled with ^111^In [79]. While this approach can be developed for clinical purposes, useful images can only be obtained for a few days after T cell infusion, owing to the half-life of ^111^In, which is 2.7 days. Real-time imaging using SPECT or positron emission tomography (PET) may also be used to monitor T cells that have been genetically modified to express appropriate reporter genes. Such an approach was initially described in pre-clinical studies using the HSV-TK gene [138]. More recently, HSV-TK has been coexpressed with a number of CARs in clinical studies, providing both a suicide gene option (see above) in addition to the opportunity to image T cells *in vivo* using either PET or SPECT. In the first such clinical study, an IL-13-based zetakine was coexpressed with HSV-TK, allowing the detection of the gene-modified T cells by PET scanning, following the administration of reporters such as (18)F-radiolabelled 9-[4-fluoro-3-(hydroxymethyl)butyl]guanine [139]. Alternative options to allow PET or SPECT imaging of T cells involve the co-expression of either the norepinephrine transporter gene [140] or the human sodium iodide symporter [141]. Both have only been used in pre-clinical studies to date but are advantaged since transgenes are human and are compatible with clinically available PET/SPECT reporters.

## 12. Clinical Trials Using First Generation CAR-Based Immunotherapy

Building upon the pre-clinical studies described above, several clinical studies have been undertaken or initiated using CAR-engineered T cells. Early studies generally involved the use of first generation CARs in which signalling was provided by CD3*ζ* alone and these are considered in turn. In some cases, studies have not been published in complete form and information has been extracted from relevant meeting abstracts.

Warren and colleagues described a CAR targeted against TAG-72 antigen which contained a CD3*ζ* endodomain [142]. From [143], it appears that up to 16 patients were treated on this study but no responses were seen. *In Vivo* persistence of T cells was not sustained; however. This finding has been repeated in several other studies, particularly those involving the use of first generation CARs in patients who did not undergo prior lymphodepletion. Six of the patients received the T cells via hepatic artery infusion. Doses of up to 10^10^ cells were used and hyperbilirubinaemia was seen in at least 2 patients.

A similar approach has also been described using a first generation CAR targeted against carcinoembryonic antigen (CEA) [143]. When last reported, 24 doses of up to 10^11^ CAR-engineered T cells were administered to seven patients with colorectal cancer and breast cancer. In two cases, IL-2 infusions were also administered. Tolerance was described as adequate. Minor responses (decreased serum CEA levels and/or reduced abdominal pain) were observed in two of the patients.

The first completed phase 1 study to be published involved a CAR with specificity for folate receptor-*α* [23]. Fourteen patients with epithelial ovarian cancer were treated with autologous T cells engineered to express a CAR in which signalling was provided by an FcR*γ* endodomain. Patients did not receive lymphodepletion in advance of infused T cells, which were administered using the IV route. The first 8 patients also received high dose IL-2 in an attempt to enhance T cell longevity *in vivo*. A different strategy was used in the final 6 patients, who received CAR-engrafted alloreactive T cells. The rationale underlying this approach was that irradiated allogeneic peripheral blood mononuclear cells could be administered to the patient in an attempt to stimulate the infused T cells *in vivo*. Grade 3-4 toxicity was observed in the initial patient cohort, but this was consistent with side effects induced by IL-2 alone. No clinical anti-tumour responses were seen in any patient. Trafficking of T cells was monitored in some patients by SPECT imaging of passively radiolabelled cells. Specific homing of T cells to tumour deposits was not observed in most cases, although, in one patient, some radiolabel did accumulate within a pelvic mass. In general, T cell survival *in vivo* was short with profound loss of T cells seen within 3 weeks in all but 1 patient. A T cell inhibitory factor (presumed to be an antibody response against the CAR) was shown to be responsible for this observation.

In the same year (2006), Lamers and colleagues described their evolving experience involving CAR-based immunotherapy of metastatic renal cell carcinoma [144]. At that time, three patients had been treated in their study with retrovirus-transduced T cells targeted against carboxy anhydrase IX (CAIX), a marker that is upregulated in tumour cells owing to hypoxia. The CAR signalling domain contained an FcR*γ* endodomain. The T cell dose was fractionated in 8 infusions over 19 days in an escalating regimen (total planned dose was 1.222 × 10^10^). Patients also received IL-2 in a fractionated regimen (5 × 10^5^ U/m^2^ on days 1–10 and 17–26). Lymphodepletion was not employed. After four T cell infusions, liver enzyme disturbances that reached grades 3-4 developed in two patients, necessitating discontinuation of treatment and institution of corticosteroid therapy in one individual. Cholangitis and unanticipated expression of target antigen was found on liver biopsy in one patient raising the strong possibility that “on-target off-tumour toxicity” was responsible. Toxicity was reversible in these patients. Circulating T cells peaked on day 6–21 and were detectable for up to 53 days by PCR. 

Five years later, an update publication described experience involving a further 8 patients on this study [24]. Patients had received a maximum of ten T cell infusions (each comprising 10^8^ cells) on days 1–5 and 29–33, in addition to IL-2. Two of the first 5 patients enrolled developed grade 3 hepatotoxicity. Consequently, the clinical trial protocol was amended so that subsequently enrolled patients received an up-front dose of anti-CAIX antibody. The underlying rationale was to achieve antigen blockade in the liver prior to infusion of T cells, in an attempt to protect from hepatotoxicity. In preliminary/unpublished data, it has been suggested that this strategy has proven effective and may also enhance *in vivo* T cell persistence. No objective clinical responses have been observed in any of the treated patients. In each case, T cells remained detectable but in declining numbers 30 days after infusions. Multiple mechanisms were uncovered that may account for this, including development of anti-idiotypic antibody and cellular immune responses directed against the CAR and also immune responses against predicted retroviral vector epitopes. 

The next published study using a first generation CAR involved 6 children with neuroblastoma [145]. Patients were treated with autologous CD8^+^ T cell clones that had been engineered by plasmid electroporation to co-express the CAR along with a hygromycin resistance gene (allowing *ex vivo* selection of transduced cells). The CAR was targeted against tumour-associated CD171 (L1 cell adhesion molecule) and contained a CD3*ζ* endodomain. T cell infusions were administered IV and in the absence of exogenous cytokine support. *in vivo* T cell persistence was generally poor (approximately 7 days after each infusion, with shortened survival observed following later infusions in some patients). One to two episodes of grade 3 toxicity were observed, including lymphopenia, neutropenia, bacteraemia, anaemia, and pneumonitis. One patient had a transient partial clinical response. Once again, the conclusion was that toxicity was acceptable but clinical efficacy inadequate. The poor survival of adoptively transferred T cells was implicated in these findings.

An innovative strategy to address the T cell survival problem was described in the next clinical trial undertaken in children with metastatic neuroblastoma [92]. In that study, investigators delivered a first generation CD3*ζ* CAR targeted against the GD2 ganglioside, either to activated T cells (ATC) or to EBV-specific cytotoxic T cells (CTL). The underlying hypothesis was that since EBV is a latent virus (and frequently reactivates subclinically), this would support the sustained survival of the CAR-engineered EBV-CTL population *in vivo*. To test this, transduced T cells from both sources were mixed in equal proportions and administered IV at total doses of 2–20 × 10^7^/m^2^. The CAR-encoding vector was identical in each case except for a noncoding oligonucleotide sequence, allowing researchers to distinguish whether CAR^+^ T cells identified *in vivo* arose from ATC or EBV-CTL. CAR engrafted CTLs reached higher levels *in vivo* than CAR engrafted ATC. Nonetheless, by 6 weeks both populations were low or undetectable. Four of the eight patients with evaluable tumours had evidence of tumour necrosis or regression, including a sustained complete remission. There were no adverse events attributable to the genetically modified T cells in the 11 subjects followed for up to 24 months.

Recently, a follow-up report on this study has been published [146]. The original 11 patients were followed for up to 5 years and 8 additional patients were recruited to the trial. Patients had active disease (*n* = 11) or no active disease (*n* = 8) at the time of treatment. The latter group had either previously relapsed or had high-risk disease. No severe or dose-limiting toxicity was observed following a total of 44 infusions (comprising transduced ATC or EBV-CTL administered to each of the 19 patients, as described above). Importantly however, low-level persistence of cells *in vivo* was demonstrated beyond six weeks by qPCR, for both T cell populations described above. Persistence was correlated with the number of CD4^+^ T cells that expressed a central memory (CD45RO CD62L^+^) phenotype. The long-term persistence of the infused cells was up to 96 weeks for CAR-CTLs and 192 weeks for CAR-ATCs. This is opposite to what was seen in the initial weeks after infusion and possibly reflects the greater proportion of central memory CD4^+^ T cells present in the ATC compared to CTL host cell populations. Clinically, persistence of CAR^+^ T cells was correlated with a greater time to disease progression in patients with active disease. Furthermore, persistence of CAR^+^ T cells at 6 weeks was found in all three of the patients who achieved complete remission (CR), having had active disease at the time of treatment). Two of these CRs were durable out to >21 and >60 months, respectively.

The first published study in which CAR-engineered T cells were tested in haematological malignancy involved 7 patients with relapsed or refractory B cell non-Hodgkin's lymphoma [97]. T cells were targeted against the CD20 antigen using an scFv-based CAR that contained a CD3*ζ* endodomain. Constructs were delivered by electroporation and cells then selected in G418, owing to a coexpressed resistance gene. Initial attempts were made to treat patients with *ex vivo* expanded T cell clones (*n* = 3) but this proved too laborious and impractical and subsequent patients were treated with G418-selected bulk cultures (*n* = 4). Following the administration of cytoreductive chemotherapy, T cells were administered to patients in 3 escalating doses (total 4.4 × 10^9^ cells) separated by 2–5 days. The last four patients also received low dose IL-2 for 2 weeks. *In Vivo* persistence of CD20-targeted T cells was observed for up to 3 weeks (in the absence of IL-2) or up to 9 weeks (with IL-2 treatment). Clinical responses were difficult to evaluate since patients also received chemotherapy, which independently exerted anti-tumour activity. No grade 3 or grade 4 toxicities were observed and no adverse events were attributed to the T cell infusions. Notably, immune responses against CAR-engineered T cells were not detected in this study, suggesting that this undesired outcome is attenuated in recipients who receive (immunosuppressive) chemotherapy or lymphodepletion.

Since this report, several other clinical trials have targeted B cell malignancy using a diversity of CAR designs. Jensen et al. reported two small studies comprising two patients each [147]. All patients received autologous T cells that had been electroporated in order to achieve CAR transgene expression and were then selected using G418 or hygromycin as appropriate. The first study entailed patients with recurrent or refractory CD20^+^ diffuse large (B) cell lymphoma who received cloned T cells that had been retargeted against CD20. Patients received T cells 28 days after an autologous haemopoietic stem cell transplant. Study two involved patients with follicular lymphoma. Polyclonal T cells were targeted against CD19 in that case. In both studies, CAR signalling was provided by CD3*ζ* and T cells were infused in the same patients in three escalating doses. In the CD19 study, fludarabine (25 mg/m^2^) was administered after the first (lowest) dose of gene-modified T cells. Cell products were generally well tolerated. Grade 2 toxicities observed included hepatotoxicity and anaemia. Grade 3 lymphopenia was also observed in 1 patient in each study and was accompanied by grade 3 eosinophilia in 1 of the patients. A self-limited febrile response with rigors (but no other features of cytokine storm such as cardiovascular instability) was observed in two patients who rejected the cells rapidly. All adverse events resolved spontaneously, without sequelae. *In Vivo* T cell persistence was short (generally up to 1 week). Both patients in the CD20 study were alive at the time of reporting although it is difficult to ascribe efficacy to cell products since other therapies were also employed. 

## 13. Clinical Trials Using Second Generation CAR-Based Immunotherapy

The initial clinical description of immunotherapy using a second generation CAR emerged in 2010 and was also focused on B cell malignancy. Kochenderfer et al. published a case report in which a patient with refractory follicular non-Hodgkin's lymphoma received T cells that expressed a CD19-targeted second generation CAR, containing a fused CD28 + CD3*ζ* signalling domain. Following preparatory lymphodepletion with cyclophosphamide and fludarabine, a dose of 4 × 10^8^ T cells was administered in a 25 : 75% split over 2 days. The patient also received high dose IL-2. A partial remission ensued that lasted 32 weeks. CAR-expressing T cells were detectable in the peripheral blood for 27 weeks but underwent exponential decay from the time of administration. No significant toxicity was observed except for transient fever and predicted on-target toxicity (e.g., depletion of normal B cells and resultant hypogammaglobulinaemia).

An updated description of the status of this study has recently been published [148]. Eight patients with advanced untreatable B cell malignancy have now been treated as above except that T cells are administered as a single dose, followed by high dose IL-2 (dose escalated until toxicity seen). Six of the eight treated patients (six of seven evaluable) had a clinical remission. The first patient was treated twice (first treatment reported in the case report above). Patient 2 died 18 days after T cell infusion with culture-proven influenza A pneumonia, nonbacterial thrombotic endocarditis and cerebral infarction and thus was not evaluable for response to treatment. Patient 3 had a sustained complete remission (durable to 15 months) with depletion of normal B cells. Patient 7 had a progressive decrease in tumour burden through to day 132 after treatment, correlating with *in vivo* persistence of CAR-engineered T cells. Trafficking of CAR^+^ T cells to bone marrow was demonstrated in some patients. B cell depletion lasting at least 6 months was observed in 4 of the 8 patients, leading to hypogammaglobulinaemia and requiring immunoglobulin replacement therapy. This was considered to be an on-target effect of the CAR-engineered T cells rather than a toxic effect of the conditioning (which generally does not cause sustained B cell depletion). Persistence of CAR^+^ T cells *in vivo* was demonstrated in peripheral blood and bone marrow and was variable in both duration and magnitude. Toxicity over the first 8 days after T cell infusion was frequent in the study, manifesting with fever, hypotension, fatigue, renal failure, and obtundation. These effects were believed to result from T cell release of IFN-*γ* and tumour necrosis factor (TNF)-*α*, following *in vivo* encounter with CD19^+^ cells (rather than resulting from exogenous IL-2 therapy or sepsis). The authors propose to modify their ongoing protocol to eliminate IL-2 infusions and will give consideration to the use of this encouraging approach after other measures designed to reduce malignant B cell burden. The anti-TNF agent etanercept was also considered as a possible therapeutic option for cytokine-mediated toxicity. A confounding variable in evaluating responses is the effect of the conditioning regimen against the disease since these agents have antilymphoma activity. Nonetheless, a direct anti-tumour effect of the infused T cells seems likely.

In parallel, Savoldo et al. performed a comparative study in which patients with B cell non-Hodgkin's lymphoma were treated with a mixture of T cells engineered to express matched CD19-specific first generation (CD3*ζ*) or second-generation (CD28 + CD3*ζ*) CARs [51]. Lymphodepletion was not used in this study. There was clear evidence of *in vivo* proliferation of the second generation CAR^+^ T cells within the initial 1-2 weeks, indicated by rising copy number in the peripheral blood. This was followed by decline to a nadir at 4–6 weeks. Comparable expansion of first generation CAR^+^ T cells did not occur. Despite this, no clinical responses were seen—two patients achieved stable disease only. No description of toxicity was provided in this study, which is a preliminary report of an ongoing trial.

The group at Memorial Sloan Kettering Cancer Center (MSKCC) are also undertaking clinical trials with a CD28 + CD3*ζ* second generation CAR targeted against CD19. Two studies are ongoing that, respectively, are recruiting patients with chronic lymphocytic leukaemia (CLL) and acute lymphoblastic leukaemia [80]. In the CLL study, 8 patients had been treated at the time of last reporting. The first 3 patients were treated in the absence of lymphodepletion at a starting T cell dose of 1.2–3.0 × 10^7^ CAR^+^ T cells/kg. No significant toxicity was encountered. However, no clinical responses were seen either and *in vivo* T cell survival was limited (i.e., engineered T cells were not conclusively demonstrated after completion of infusion). Consequently, the investigators moved to the next phase of the study, which involved prior lymphodepletion with cyclophosphamide 1.5 g/m^2^ followed by infusion of the same T cell dose. The first patient treated in this cohort was a 69-year-old male with bulky CLL and with a past medical history of ischaemic heart disease, hypertension, and chronic renal impairment. After the T cell infusion, he developed fever. This had been seen as a transient phenomenon in earlier treated patients but was persistent in this case. This was followed by hypotension, renal failure, then multiorgan failure, and ultimately death 44 hours after the infusion [88]. Notably, serum cytokine levels in that patient were markedly elevated in samples taken following administration of cyclophosphamide but prior to adoptive T cell transfer. Sterility of the T cell product was confirmed after death. At post mortem, no significant pathology was found in lungs, heart, or kidneys and there was no evidence of tumour lysis syndrome. The investigators modified their protocol thereafter so that serum cytokines were measured in all subjects. However, they never again encountered the situation where cytokine levels were elevated prior to T cell infusion. In retrospect, it is believed that the cause of death was subclinical sepsis that was aggravated by cyclophosphamide-mediated immunosuppression. Following this suspected unexpected serious adverse reaction (SUSAR), subsequent patients were treated at a −1 dose level (0.4–1 × 10^7^ CAR^+^ T cells/kg) administered as a split infusion after cyclophosphamide-induced lymphodepletion. No further severe toxic episodes were seen in patients subsequently enrolled in the study (*n* = 5). 

Other toxicity has been described in these ongoing studies at MSKCC as follows. Fever with or without rigors was observed following T cell infusion in all patients. Although patients were empirically treated with antibiotics (since they were highly immune compromised), these were stopped once cultures were found to be sterile. It seems in retrospect that these side effects were directly attributable to the infused T cells and are consistent with toxicity reported in the study of Kochenderfer et al. [148]. Second, persistent B cell aplasia has been observed in the single treated patient with ALL. The single patient with ALL had sustained B cell aplasia. Most likely, this is due to on-target toxicity mediated by the engineered T cells, although reinduction chemotherapy had also been administered. Third, one patient developed transient hypotension after infusion of T cells, a finding that was reversed with increased IV fluids.

A number of clinical responses have been observed in this study to date. The second CLL patient treated at the −1 dose had a partial remission. Two subsequent patients have had stable disease. Patients treated in this cohort were found to have detectable CAR^+^ T cells in bone marrow 6 weeks after the T cell infusion was administered.

Evidence that T cells could traffic to sites of disease was also provided. Gene-modified T cells could be demonstrated both in bone marrow and lymph nodes at sites of disease in the patient who died of presumed sepsis, shortly after infusion of T cells. 

One unusual attribute of the T cell products generated in this study was the preponderance of CD4^+^ T cells in the final products. The reason for this is unclear. There were very few regulatory T cells contained therein but nonetheless this skewed CD4/CD8 ratio within cell products may conceivably have compromised anti-tumour activity.

Investigators at the University of Pennsylvania are also treating patients with CD19^+^ malignancy using a related but distinctive CAR-based immunotherapeutic strategy. This group is also using a second generation CAR but in which co-stimulation is provided by 4-1BB (CD137) instead of CD28. To date, they have reported on three patients with advanced CLL, all of whom received lymphodepleting chemotherapy prior to infusion of T cells [49, 50]. Two of the three patients have achieved a complete and sustained clinical remission. The third patient achieved partial remission and was unique in that circulating tumour cells were present achieved partial remission. This study is the most successful and remarkable example of the promise associated with CAR-based immunotherapy. What sets this study apart from all others is the pronounced proliferation of T cells that occurred *in vivo* in all three treated patients. Patients did not receive IL-2. A possible explanation for these findings is the use of 4-1BB rather than CD28 as the co-stimulatory motif within the CAR. In pre-clinical studies that evaluated this CAR, there was a clear evidence that the 4-1BB CAR enabled activated T cells to proliferate even in the absence of CAR engagement [47]. This has never been observed using first generation or CD28-CD3*ζ* second-generation CAR designs. A further contributory factor that may have supported the T cells *in vivo* is the fact that B cells are under constant production in the bone marrow, providing an ongoing “antigenic” stimulus to the infused cells. Transduced T cells persisted in the bloodstream of all patients for at least 6 months.

The most striking of these three responses occurred in a patient who received a dose of 3 × 10^8^ T cells, of which only 5% were transduced. Transduction efficiency (using a lentiviral vector) was below the pre-set specification for cell product release but the patient was treated nonetheless following approval by the local Institutional Review Board. The infused T cells subsequently expanded by 3 logs *in vivo* and caused delayed tumour lysis syndrome followed by a complete, sustained and durable clinical remission [50]. Anecdotes such as this indicate the much remains to be learned about appropriate dosing using CAR-engineered T cells.

Toxicity was also observed in this study but was acceptable. Transient and mild febrile reactions occurred within the first 4 days after the T cells were administered. However, all patients developed a clinical and laboratory evidence of toxicity within 7–21 days of the 1st infusion (the total dose was divided over 3 days). These adverse events included high fever, rigors, transient hypotension, and dyspnoea, requiring hospitalization of 1 patient. Toxicity correlated with the fact that infused T cells had undergone massive *in vivo* proliferation leading to a delayed and profound clearance of tumour. 

## 14. Clinical Trials Using Third Generation CAR-Based Immunotherapy

In the light of the promise shown in trials using second generation CARs, it is not surprising that investigators have begun to explore third generation fusions in which two co-stimulatory domains are present. Till et al. have reported preliminary results in which four patients with mantle cell or follicular non-Hodgkin's lymphoma were treated with T cells that expressed a CD20-specific CAR, containing a fused CD28/4-1BB/CD3*ζ* endodomain [149]. Gene transfer was achieved by electroporation of plasmid DNA (containing a G418 selection marker, meaning that prolonged *ex vivo* culture in G418 was required). CAR expression was undetectable at the protein level but could be detected by PCR. Patients received T cells in three divided doses of 10^8^, 10^9^ and 3 × 10^9^/m^2^ separated by 2–5 days and followed by 14 days of IL-2 (250,000 IU/m^2^ by the subcutaneous route, twice daily). Infusions were generally well tolerated although one elderly patient had fever, hypoxaemia, and hypotension after infusions 2 and 3. Three of four patients received moderate intensity lymphodepletion with cyclophosphamide 1 g/m^2^ two days before initiation of the T cell infusions. Two patients without evaluable disease remained progression free for 12 and 24 months. The third patient had an objective partial remission and relapsed at 12 months after infusions.

A study involving a third generation CAR was also instigated in patients with solid tumours, targeted against HER2 (ErbB2) [89]. The chimeric antigen receptor consisted of (subnanomolar affinity) HER2-specific trastuzumab scFv, coupled to a fused CD28/4-1BB/CD3*ζ* trimodular endodomain. The first patient enrolled was a 39-year-old female patient with metastatic HER2^+^ colorectal cancer. The patient received lymphodepleting chemotherapy followed by at least 10^10^ engineered T cells, which were 79% CAR^+^ and were infused intravenously over 30 minutes. Unfortunately however, the patient succumbed to a fatal serious adverse event, which was clearly attributable to the infused T cells. Respiratory distress commenced within 15 minutes of completion of the T cell infusion, followed by progressive pulmonary oedema and death within 5 days. At autopsy, adult respiratory distress syndrome, multiorgan and systemic microangiopathy, rhabdomyolysis, and gastrointestinal haemorrhage were evident. However, there was no evidence that T cells had trafficked in a specific manner to tumour deposits. Serial blood sampling during the terminal event confirmed that a cytokine storm had also occurred, the onset of which was evident within 4 hours of T cell infusion. The cause of death has variously been ascribed to immune recognition of the HER2 target either within the pulmonary parenchyma or microvasculature [150].

## 15. Ongoing and Unpublished Clinical Trial Activity

In addition to the studies described in the foregoing sections, a number of unpublished clinical trials of CAR-based immunotherapy are ongoing and these are listed in Table 3.

## 16. Conclusions and Future Perspectives

Immunotherapy using CAR-engineered T cells has come a long way and is now bearing fruit in the clinic, although significant concerns remain regarding potential for unpredictable acute and chronic toxicity. Several obstacles have been identified that have hampered the effectiveness of this therapy, particularly in the context of solid tumours. Ideally, systems are required whereby T cells are engineered to undergo maximal activation upon engagement of one or more target molecules the expression profile of which demarcates clearly between tumour cells and their healthy counterparts. Smaller cell doses are desirable for reasons of safety and practicality. This may prove possible if systems can be devised to enable T cells to expand *in vivo* in a controlled manner, to persist *in vivo* for longer, to home to tumour deposits effectively and to thrive in the hostile tumour microenvironment, persisting there in a functionally competent state that is dependent upon the ongoing presence of tumour cells. To maximize therapeutic benefit, it is likely that future CAR-based immunotherapies will also need to activate other strands of the host immune response. This may include the harnessing of innate immune effector mechanisms and/or the induction or amplification of adaptive immune responses directed against *de facto* tumour antigens. In parallel, there is a need to develop more robust, practical, and automated systems to generate stable cell products so that these therapies become more accessible, feasible, and economical to produce. The challenge of commercializing cell therapy also remains an issue, although there have been encouraging recent developments. Ultimately, an iterative cycle from bench to clinic and back again is required to bring these therapies to full maturity.

## Figures and Tables

**Table 1:** Targets for CAR-based immunotherapy.

Target	Malignancies	Nature of antigen	Selected references
CD19	B cell	Protein	[39, 44, 151–153]
CD20	B cell	Protein	[154]
CD22	B cell	Protein	[27]
k light chain	B cell	Protein	[155]
CD30	Hodgkin's and non-Hodgkin's lymphomas	Protein	[82, 156]
CD33	Myeloid	Protein	[94, 157]
CD123	Myeloid	Protein	[158]
CD38	B cell	Protein	[159, 160]
ROR1	B cell	Protein	[161]
ErbB2	Several, including breast, osteosarcoma, prostate, medulloblastoma, glioblastoma	Protein	[11, 21, 25, 35, 85, 162–173]
ErbB3/4	Several	Protein	[11, 12]
Several ErbB dimers	Several	Protein	[14]
EGFr vIII	Several	Protein	[174]
Carcinoembryonic antigen	Several	Protein	[31, 36, 175]
EGP2	Several	Protein	[176]
EGP40	Colon	Protein	[177]
Mesothelin	Several	Protein	[37, 178]
TAG72	Gastrointestinal	Carbohydrate	[179]
PSMA	Prostate; tumour-associated neovasculature	Protein	[34, 180]
NKG2D ligands	Several	Protein	[181]
B7-H6	Several	Protein	[16]
IL-13 receptor *α*2	Several	Protein	[136, 182, 183]
MUC1	Breast, ovarian	Heavily glycosylated protein	[22]
MUC16	Ovarian	Heavily glycosylated protein	[184]
CA9	Renal cell carcinoma	Protein	[185]
GD2	Neuroblastoma, Ewing's sarcoma	Ganglioside	[32, 186, 187]
GD3	Melanoma	Ganglioside	[188]
HMW-MAA	Melanoma	Proteoglycan	[189]
CD171	Neuroblastoma	Protein	[145]
Lewis Y	Several	Carbohydrate	[190]
G250/CAIX	Renal cell carcinoma	Protein	[144]
HLA-A1 MAGE A1	Melanoma	Protein-Peptide complex	[4]
HLA-A2 NY-ESO-1	Several	Protein-Peptide complex	[191]
PSCA	Prostate	Protein	[192]
Folate receptor-*α*	Ovarian and others	Protein	[193, 194]
CD44v6	Several	Protein	[195]
CD44v7/8	Cervical	Protein	[196]
*α*_ v_ *β*_ 6_ integrin	Several	Protein	[13]
8H9	Several	Protein	[197]
NCAM	Neuroblastoma	Protein	[198]
VEGF receptors	Several	Protein	[199, 200]
5T4	Several	Protein	[201]
Foetal AChR	Rhabdomyosarcoma	Protein	[202]
NKG2D ligands	Several	Protein	[72]
CD44v6	Several	Protein	[203]
Dual antigen	Activation by cells that express either targets	Any	[204]
Dual antigen	Maximal activation when both targets expressed	Any	[52]
Universal	All	Any	[18, 19]

AChR: acetylcholine receptor; CA9: carbonic anhydrase 9; EGFr: epidermal growth factor receptor; EGP: epithelial glycoprotein; GD: ganglioside; HWM-MAA: high molecular weight melanoma-associated antigen; MUC: mucin; NCAM: nerve cell adhesion molecule; NKG2D: natural killer group 2 member D; PSCA: prostate stem cell antigen; PSMA: prostate-specific membrane antigen; ROR1: Receptor-tyrosine-kinase-like orphan receptor 1; TAG: tumour-associated glycoprotein; VEGF: vascular endothelial growth factor.

**Table 2:** Host cells other than autologous T-cells used for CAR-based immunotherapy.

Target	Cellular host	Reference
ErbB2	NK92 cells	[205–207]
ErbB2	Primary NK cells	[208, 209]
ErbB2	T-cells and NK cells	[117]
CEA	Monocytes	[210]
CD19	Umbilical cord blood T cells	[211]
CD19/GD2	*γδ* T cells	[68]
CD19	Allogeneic T cells	[69]
CD19	NK cells	[64, 65]
Human immunodeficiency Virus GP120	Neutrophils	[212]
Folate-binding protein	Haematopoietic stem cells	[213]
ErbB2	Dendritic cells	[214]

**Table 3:** Ongoing unpublished trials.

Target	Identifier	Institution	CAR generation	Disease	Comments
CD19	NCT01087294	National Cancer Institute	?	B cell malignancy	
HER2	NCT00902044	Baylor College of Medicine	2	Sarcoma	
K light chain	NCT00881920	Baylor College of Medicine	1/2	B cell/ myeloma	
HER2	NCT01109095	Baylor College of Medicine	2	GBM	Autologous CMV CTLs
CD19	NCT00924326	National Cancer Institute	2	B cell	
CD19	NCT01475058	Fred Hutchinson Cancer Research Center	2	B cell	
CD30	NCT01316146	Baylor College of Medicine	2	Hodgkins/NHL	
CD30	NCT01192464	Baylor College of Medicine	1	CD30+ lymphoma	Autologous EBV CTLs
EGFRvIII	NCT01454596	National Cancer Institute	3	Glioma	
CD19	NCT01318317	City of Hope Medical Center	?	B cell	
HER2 (plus TGF*β* Dominant negative receptor)	NCT00889954	Baylor College of Medicine	2	HER2 positive	
CD19	NCT01593696	National Cancer Institute	2	B cell malignancy	
GD2	NCT01460901	Children's Mercy Hospital, Kansas City	?	Neuroblastoma	
CD19	NCT01430390	Memorial Sloan Kettering Cancer Center	?	ALL	Donor EBV CTLs post-BMT
CD19	NCT01493453	Christie Hospital NHS Foundation Trust	1	B cell malignancy	Suspended
CEA	NCT01212887	Christie Hospital NHS Foundation Trust	1	Multiple	
CD19	NCT01044069	Memorial Sloan Kettering Cancer Center	2	Pre-B-ALL	
CD19	NCT00840853	Baylor College of Medicine	2	B-cell malignancy	Virus-specific CTL [215]
CD19	NCT00586391	Baylor College of Medicine	1 and 2	B-cell malignancy	
CD19	NCT00709033	Baylor College of Medicine	2	B-cell malignancy	
PSMA	NCT01140373	Memorial Sloan Kettering Cancer Center	2	Prostate cancer	HSV-TK suicide gene [216]
PSMA	NCT00664196	Roger Williams Medical Center	?	Prostate cancer	[217]
Mesothelin	NCT01355965	University of Pennsylvania	2 (4-1BB)	Mesothelioma	Lentiviral vector
CD19	NCT01029366	University of Pennsylvania	2 (4-1BB)	B-cell malignancy	Lentiviral vector
CD19	NCT01195480	University College London	1	B-ALL	Donor EBV-CTL post-BMT
IL-13R*α*2 and HSV-TK	NCT01082926	City of Hope Medical Center	1	Glioma	PET Imaging
CD19	NCT01362452	MD Anderson Cancer Center	?	B-cell malignancy	Donor derived after UCBT
Extended ErbB family	EudraCT 2012-001654-25	King's College London	2	Head and neck Cancer	Intratumoral delivery
Folate receptor-*α*	Not known	University of Pennsylvania	2 (4-1BB)	Ovarian cancer	Lentiviral vector [218]

Search terms chimeric antigen receptor; chimeric T cell or zeta on 8.9.2012.

BMT: bone marrow transplantation; CTL: cytotoxic T cell; CMV: cytomegalovirus; EBV: Epstein Barr virus; HSV-TK: herpes simplex virus thymidine kinase; PET: positron emission tomography; PSMA: prostate-specific membrane antigen; UCBT: umbilical cord blood transplantation.
